# Longitudinal Analysis of QuantiFERON-TB Gold In-Tube in Children with Adult Household Tuberculosis Contact in South Africa: A Prospective Cohort Study

**DOI:** 10.1371/journal.pone.0026787

**Published:** 2011-10-31

**Authors:** Maunank Shah, Tafadzwa S. Kasambira, Peter V. Adrian, Shabir A. Madhi, Neil A. Martinson, Susan E. Dorman

**Affiliations:** 1 Johns Hopkins University School of Medicine, Baltimore, Maryland, United States of America; 2 Respiratory and Meningeal Pathogens Research Unit and Department of Science and Technology National Research Foundation: Vaccine Preventable Diseases, University of Witwatersrand, Johannesburg, South Africa; 3 Perinatal HIV Research Unit, University of Witwatersrand, Johannesburg, South Africa; National Institute for Infectious Diseases (L. Spallanzani), Italy

## Abstract

**Background:**

QuantiFERON-TB Gold In Tube (QFT-GIT) is a tool for detecting *M. tuberculosis* infection. However, interpretation and utility of serial QFT-GIT testing of pediatric tuberculosis (TB) contacts is not well understood. We compared TB prevalence between baseline and 6 months follow-up using QFT-GIT and tuberculin skin testing (TST) in children who were household contacts of adults with pulmonary TB in South Africa, and explored factors associated with QFT-GIT conversions and reversions.

**Method:**

Prospective study with six month longitudinal follow-up.

**Results:**

Among 270 enrolled pediatric contacts, 196 (73%) underwent 6-month follow-up testing. The 6-month prevalence estimate of MTB infection in pediatric contacts increased significantly from a baseline of 29% (79/270, 95%CI [24–35]) to 38% (103/270, 95% CI [32–44], p<0.001) using QFT-GIT; prevalence increased from a baseline of 28% (71/254, 95%CI [23–34]) to 33% (88/263, 95%CI [21–32], p = 0.002) using TST. Prevalence estimates were influenced by thresholds for positivity for TST, but not for QFT-GIT. Among 134 children with a negative or indeterminate baseline QFT-GIT, 24 (18%) converted to positive at follow-up; conversion rates did not differ significantly when using more stringent thresholds to define QFT-GIT conversion. Older age >10 years (AOR 8.9 95%CI [1.1–72]) and baseline TST positivity ≥5 mm (AOR 5.2 95%CI [1.2–23]) were associated with QFT-GIT conversion. Among 62 children with a positive baseline QFT-GIT, 9 (15%) reverted to negative; female gender (AOR 18.5 95%CI [1.1–321]; p = 0.04] was associated with reversion, while children with baseline positive TST were less likely to have QFT-GIT reversion (AOR 0.01 95%CI [0.001–0.24]).

**Conclusion:**

Among pediatric contacts of adult household TB cases in South Africa, prevalence estimates of TB infection increased significantly from baseline to 6 months. Conversions and reversions occurred among pediatric TB contacts using QFT-GIT, but QFT-GIT conversion rates were less influenced by thresholds used for conversions than were TST conversion rates.

## Introduction

In the wake of a significant HIV epidemic, South Africa has experienced a dramatic rise in the incidence of tuberculosis (TB) [1]. Children are particularly vulnerable, as they have high rates of progression from latent tuberculosis infection (LTBI) to active TB disease and are at risk for severe forms of TB disease [2], [3]. These issues underscore the need for early detection and diagnosis of *M. tuberculosis* (MTB) infection in children. Childhood tuberculosis is usually a consequence of recent transmission from an adult with pulmonary TB [4]. Therefore, early identification of TB infection among childhood contacts of adults with pulmonary TB may be important to prevent rapid progression to active TB in TB endemic settings. Active contact tracing of children who are household contacts of adult TB cases is currently recommended in South Africa [5]. However, the optimal test and testing strategy for detecting TB infection in pediatric contacts is unknown.

Tuberculin skin testing (TST) is the most widely used method for detecting TB infection, but has limitations. There can be immunological cross-reactivity between TST reagents and Bacille Calmette Guerin (BCG) vaccine, thereby complicating the interpretation of TST results in areas where BCG is administered. Interferon-gamma (IFN-γ) release assays (IGRAs), such as the commercially available QuantiFERON-TB Gold In Tube (QFT-GIT, Cellestis, Ltd, Carnegie, Australia) and T-SPOT.TB tests (Oxford Immunotec Limited, Abingdon, United Kingdom), have the potential to overcome some of TST's limitations [6]. IGRAs detect MTB infection by measuring *in vitro* IFN-γ release following stimulation of lymphocytes with antigens specific to *M. tuberculosis*, and offer the theoretical advantage of higher specificity in BCG-vaccinated populations [6]. In contrast to TST, IGRAs require only one visit to obtain a result, making them attractive for use in contact investigations. Some guidelines endorse the use of IGRAs as alternatives to TST for detection of TB infection, but studies evaluating IGRA performance in children are lacking [7].

Several issues about the use of IGRAs in pediatric TB contact investigations remain unresolved. The timing of IGRA conversion after new infection is not clear, and some have recommended serial testing when used as part of contact investigations [7]. The performance of IGRAs in serial testing in children has not been fully explored, although adult studies suggest high rates of apparent conversions and reversions [8]. Given potential within-person variability in test results, the optimal threshold to define IGRA conversion is unclear, since minor variations around the diagnostic cut-off points could lead to misclassifications [9]. It is unclear whether the same cut-off that is used for initial diagnosis of LTBI (≥0.35 IU/ml or ≥8spots for QFT-GIT and T-SPOT.TB, respectively) should also be used to define a test conversion on serial testing [8], [9], [10].

The yield of serial testing for contact investigations in pediatric populations after diagnosis of an adult household case in South Africa and other TB-prevalent settings is unknown. Early immunologic-based testing of pediatric contacts soon after TB infection may not allow sufficient time for immunologic responses to develop, leading to an underestimation of true TB prevalence. Moreover, there may be ongoing household TB transmission that would be missed on initial TB investigations. Follow-up TB testing of pediatric contacts may thus identify additional cases of TB infection, but the yield of such a strategy in South Africa has not been fully explored.

We previously reported a high prevalence of MTB infection as assessed by a single TST (prevalence of 28% using a 5 mm cut-off) or QFT-GIT test (prevalence 29%), among children in South Africa who were household contacts of an adult with newly diagnosed pulmonary TB [11]. We now explore and compare the yield of performing a 6-month follow-up TST and QFT-GIT in detecting TB infection among these children. We also examined the dynamics of TST and QFT-GIT responses on serial testing in children, and evaluated the impact of using varying thresholds for defining test conversions.

## Methods

### Ethics Statement

The study was approved by ethics committees at the University of the Witwatersrand (Johannesburg, South Africa) and the Johns Hopkins University School of Medicine (Baltimore, USA). Written informed parental consent (and assent for children aged ≥7 years) was obtained from all participants.

### Objectives

We sought to determine the 6-month prevalence of *M. tuberculosis* infection among children who were household contacts of adult pulmonary TB cases, and to compare baseline prevalence estimates to 6 month testing results. We also sought to compare the prevalence estimates as determined by two different tests for TB infection, the TST and QFT-GIT tests, and to identify factors associated with prevalent pediatric TB infection. We explored the dynamics of serial testing by exploring different thresholds to define TST and QFT-GIT conversion, and by examining factors associated with test conversions and reversions.

### Participants

Study methods for baseline TB testing have been described in detail previously [11]. Briefly, adult pulmonary TB index cases and children living in Soweto, South Africa between October 2006 and December 2009 were enrolled. Index adult TB cases aged ≥18 years who had been diagnosed with pulmonary TB within the preceding three months and had at least one age-eligible child in the household were study eligible. Pediatric contacts of enrolled adult TB index cases were included if they were age ≥6 months to ≤16 years; children were excluded if they had a prior diagnosis or treatment of active or latent TB.

### Description of Procedures

For pediatric contacts, demographic, medical, and TB exposure and treatment information was collected through interview of the enrolled parent/guardian. TST and QFT-GIT were performed at the time of enrollment on all pediatric contacts at the same study visit; phlebotomy for QFT-GIT was performed immediately after TST placement. For TST, tuberculin purified protein derivative (PPD) RT-23 (2 units, Statens Serum Institut, Copenhagen, Denmark) was injected subcutaneously into the left forearm and the test was read 48–72 hours later. An induration of ≥5 mm was considered a positive TST during the study, as per ATS/CDC/IDSA TB guidelines for TB contacts [12]. QFT-GIT testing was performed according to the manufacturer's instructions [13]. The QFT-GIT assay included a nil control, mitogen control, and an antigen tube. All assays were conducted in a single laboratory by the same trained technician. Following stimulation and centrifugation, harvested plasma specimens were stored at 4°C for up to 28 days prior to ELISA testing. Results were calculated and interpreted by the assay software as positive, negative, or indeterminate, according to manufacturer's instructions [13]. HIV testing using an age-appropriate test was performed at the baseline visit on pediatric contacts.

Children had a follow-up study evaluation at 6 months after their initial visit. Interval medical history and TB exposure/treatment history were obtained through interview with the parent/guardian. All children received a QFT-GIT at the 6-month follow-up visit. Children with a baseline positive TST (≥5 mm induration) did not have a repeat TST performed. Children with a negative baseline TST (<5 mm) had a repeat TST performed. A TST of ≥5 mm induration at 6 month follow-up was considered positive during study implementation. Children meeting any of the following criteria at baseline or follow-up were referred to local sources of medical care: signs and/or symptoms of active TB, TST≥5 mm and/or positive QFT-GIT (as interpreted by assay software), positive HIV test, or age <5 years.

### Statistical considerations

Prevalence of MTB infection was defined as having a positive test result at baseline and/or 6 months by TST or QFT-GIT. For the primary analysis and from an operational perspective for study implementation, a TST conversion at 6 months was defined as having a negative TST at baseline, with a TST≥5 mm induration at 6 month follow-up. QFT-GIT conversion was defined as having a negative or indeterminate QFT-GIT at baseline, with a positive QFT-GIT at 6 months follow-up. South African guidelines currently suggest a TST≥10 mm is positive in children. They note that thresholds between 5–14 mm can be considered if clinical history suggests contact with an active TB case [5]. Secondary analyses thus explored the following additional definitions of TST conversion: 1) baseline TST<5 mm, and follow-up TST≥5 mm induration (least stringent); 2) baseline TST<10 mm and follow-up TST≥10 mm induration (more stringent absolute cutoff); 3) baseline TST<5 mm and follow-up TST increase by at least 10 mm induration (most stringent). Secondary analyses also explored the following four definitions for QFT-GIT conversion that have been previously proposed in the literature [8]: 1) baseline IFN-γ <0.35 IU/ml and follow-up IFN-γ ≥0.35 IU/ml (i.e. negative to positive change according to manufacturer's criteria; least stringent); 2) baseline IFN-γ <0.35 IU/ml and follow-up IFN-γ ≥0.35 IU/ml, plus at least a 30% increase in IFN-γ over baseline value; 3) baseline IFN-γ <0.35 IU/ml and follow-up IFN-γ ≥0.35 IU/ml, plus at least an absolute 0.35 IU/ml increase in IFN-γ over baseline value; 4) follow-up IFN-γ ≥0.70 IU/ml (most stringent). Concordance between QFT-GIT and TST results was assessed using the kappa statistic. Categorical data were compared using χ^2^ and McNemar's tests. Factors associated with QFT-GIT and TST results were assessed using univariate and multivariate logistic regression analysis, with robust variance and clustering on index cases. Covariates included in multivariate analysis were based on stepwise selection (p<0.3) or clinical importance (i.e. age, gender, HIV status, index case TB status) [14]. Regression analyses were performed separately for index case factors and pediatric factors. Data were analyzed using STATA (version 10.1, StataCorp, College Station, Texas).

## Results

### Study profile and baseline testing

A total of 169 adult index TB cases and 270 pediatric contacts were enrolled with baseline characteristics shown in Table 1. Results of baseline testing have been described previously [11]. Briefly, the estimated prevalence of MTB infection at baseline was similar between QFT-GIT and TST when a 5 mm induration cut-off for TST positivity was used (29% [79/270] vs. 28% [71/254] for QFT-GIT and TST, respectively [p = 0.49, kappa 0.58]). When a baseline TST induration of ≥10 mm was considered positive, the estimated MTB prevalence differed significantly between the two tests (22% [57/254] vs. 29%, for TST and QFT-GIT respectively; p = 0.002; kappa 0.54).

**Table 1:** Characteristics of study participants at enrollment and follow-up.

Characteristic		Value, as n (%) unless otherwise specified			
		Baseline Pediatric Contacts N = 270	Pediatric Contacts with 6 month Follow-up N = 296	Pediatric Contacts Lost to Follow-up N = 74	p**
Sex	Male n(%)	129 (48)	92(47)	37(50)	.65
Median age (IQR)		6 (3–9)	6(3–9)	6(4–10)	.23
Ethnicity, n(%)	African/Black	256 (95)	123(95)	39(98)	.21
	Colored/Mixed	11 (4)	5(4)	1(3)	
	Unspecified or Other	3(1)	2(2)	0(0)	
HIV, n(%)	Infected	14 (5)	7(4)	7(9)	.035
	Uninfected	251 (93)	187 (95)	64(87)	
	Unknown	5 (2)	2(1)	3(4)	
Median weight for age Z score (IQR)*		0.13 (−0.86 to 0.97)	.11 (−.83 to.93)	.25(−.86 to 1.21)	.89
Median weight for height Z score (IQR)*		1.3 (−0.05 to 2.14)	0.94 (−.3 to 2.3)	1.3 (.09 to 2.1)	.99
Median length for age Z score (IQR)*		−1.44 (−2.8 to −0.5)	−1.2 (−2.7 to −.7)	−1.6 (−2.9 to −.37)	.40
BCG vaccinated per report of parent/guardian, n(%)		257 (95)	186 (95)	71(96%)	.38
Baseline positive TST (≥5 mm), n%		71/254† (28)	60/185† (32)	11/69† (16)	.009
Baseline positive QFT-GIT, n%		79 (29)	62 (32)	17 (23)	.163

Abbreviations: IQR, interquarterile ratio.

*for children ≤60 months old.

**comparing pediatric contacts with follow-up vs. contacts that were lost.

† 16 pediatric contacts did not have baseline TST results.

A 6-month follow-up visit was completed for 196/270 (72.5%) children, and the remainder were lost to follow-up (Figure S1). There was no difference in median age, gender, nutritional status, or BCG status between children with a follow-up visit and those that lost to follow-up (Table 1). Similarly, there was no difference in baseline QFT-GIT positivity rates between those that had a follow-up visit (32% QFT-GIT positive), and those lost to follow-up (23% QFT-GIT positive; p = 0.163), though baseline TST positivity rates were higher among children with follow-up (32% TST positive) compared to those who were lost (16% TST positive; p = 0.009). While overall rates of HIV were low in our study, there was a slightly higher rate of HIV infection among those children that were lost to follow-up (7/74 [9%]) compared to those with a 6 month visit (7/196[4%]; p = 0.035).

### Prevalence of TB infection in pediatric contacts at 6 months follow-up by QFT-GIT and TST

Among the 196 children who had a follow-up visit, all 196 (100%) had a repeat QFT-GIT performed, while 127 (65%) had a TST completed at 6 months (60 children had baseline positive TST [≥5 mm] and did not have a second TST per the study protocol). All 270 pediatric contacts had either a baseline and/or follow-up QFT-GIT testing performed, while 263 children had either a baseline and/or 6 month follow-up TST performed (Figure S1).

The prevalence of MTB infection as ascertained by QFT-GIT increased significantly from a baseline of 29% (79/270; 95% CI [24–35]) to 38% (103/270, 95%CI [32–44], p<0.001; Table 2) at six months of follow-up. There was no difference in the six-month MTB period prevalence estimate when using more stringent thresholds for QFT-GIT conversion (Table 2). If only the 196 children with follow-up visits were included in the analysis, the period prevalence of MTB infection would rise from a baseline 32% (62/196, 95% CI 25–39]) to 44% (86/196,95% CI [37–51], p<0.01) six months later.

**Table 2:** Prevalence of TB infection and incidence of conversions using QFT-GIT.

	Prevalence of TB infection†			Conversions	
QFT-GIT thresholds for baseline positivity and conversion at follow-up testing	Baseline n (%)	6 month n (%)	p values †††	Negative or indeterminateBaseline QFT-GIT *n*	Conversions *n* (%)
Baseline or follow-up QFT-GIT ≥0.35 IU/ml	79/270 (29%)	103/270 (38%)††	p<0.01	134	24 (18%)°
Baseline QFT-GIT ≥0.35 IU/ml or Follow-up QFT-GIT ≥0.35 IU/ml, plus 30% increase over baseline	79/270 (29%)	103/270 (38%)††	p<0.01	134	24 (18%)°
Baseline QFT-GIT ≥0.35 IU/ml or Follow-up QFT-GIT ≥0.35 IU/ml, plus absolute increase of 0.35 IU/ml over baseline	79/270 (29%)	102/270 (38%)††	p<0.01	134	23 (17%)°
Baseline QFT-GIT ≥0.35 IU/ml or Follow-up QFT-GIT ≥0.70 IU/ml	79/270 (29%)	102/270 (38%)††	p<0.01	134	23 (17%)°

† 6-month prevalence defined as a positive QFT-GIT result, at either baseline and/or follow-up. 270 children had QFT-GIT testing at baseline. 6 month QFT-GIT testing was performed on 196 children; 134 had negative or indeterminate baseline QFT-GIT tests and were included in analysis of conversions. 74 children were lost to follow-up.

†† p>0.05 for all pair-wise comparisons.

††† p values compare rate of baseline positivity to follow-up.

° p>0.05 for all pair-wise comparisons; if only those with baseline negative results are included (i.e. exclude indeterminates), the rates of QFT-GIT conversion for all thresholds was 22/117 (18.8%,).

The prevalence of MTB infection as ascertained by TST (TST≥5 mm induration) in pediatric contacts increased significantly from a baseline of 28% (71/254, 95%CI [23–34]) to 33% (88/263, 95%CI [21–32], p.002; Table 3) six months later. The increase in estimated prevalence from baseline to follow-up was seen even when using higher thresholds for TST positivity (Table 3). When a threshold of TST≥10 mm induration was considered, the MTB prevalence increased from a baseline of 22% (57/254, 95% CI [17–28]) to 27% (70/263, 95% CI [21–32], p<0.001). When an even higher threshold for TST conversion was considered (i.e. absolute TST increase in 10 mm from baseline to follow-up), there was still a significant increase in 6-month MTB prevalence from a baseline of 28% (71/254, 95%CI [23–34]) to 31% (81/263 95%CI [25–37]), p<0.01). Despite these increases, however, the overall MTB period prevalence estimates at 6 months were significantly lower when using the higher threshold of TST≥10 mm (27%) or a threshold requiring increase in 10 mm induration (31%), compared to using a 5 mm cut-off (33%,Table 3). If only the children with follow-up visits were included in the analysis, the estimated prevalence of MTB infection ascertained by TST (TST≥5 mm induration) would rise from 32% (60/185, 95%CI [26–40]) to 40% (77/194, 95%CI [33–47], p<0.01).

**Table 3:** Prevalence of TB infection and incidence of conversions using TST.

	Prevalence of TB infection†			Conversions	
TST Cutoffs for baseline positivity and conversion	Baseline n (%)	6 month n (%)	p values ††	Negative Baseline TST *n*	Conversions *n* (%)
TST≥5 mm at baseline or follow-up	71/254 (28%)	88/263 (33%)°	p<0.01	127	17 (13%)**
TST≥10 mm at baseline or follow-up*	57/254 (22%)*	70/263 (27%)* °	p<0.01	127	13 (10%)**
Baseline TST≥5 mm; Follow-up TST increase from baseline of ≥10 mm	71/254 (28%)	81/263 (31%)°	p<0.01	127	10 (8%)**

† 6-month prevalence defined as a positive TST result, at either baseline and/or follow-up. 254 children had TST testing at baseline. Among the 16 children without baseline TST testing, 9 had follow up testing; 263 children had a TST performed at either baseline or follow-up. 6 month TST testing was performed on 127 children who had baseline negative TST results and were included in analysis of conversions (includes 9 children without baseline TST results). 74 children were lost to follow-up.

†† p values compare rate of baseline positivity to follow-up.

*14 children had baseline TST induration between 5 mm and 10 mm and were classified as negative using a threshold of TST≥10 mm; these children did not receive follow-up TST per study protocol based on having TST≥5 mm.

° Pair-wise comparisons were made between TST thresholds. 6 month prevalence comparing TST threshold of ≥5 mm to threshold of TST≥10 mm: p<0.001; comparing a threshold of TST increase of ≥10 mm to a threshold of ≥5 mm: p = 0.02; comparing threshold of TST increase of ≥10 mm to absolute threshold of ≥10 mm: p = 0.01.

**Pair-wise comparisons were made between TST thresholds. Conversion rates comparing TST threshold of ≥5 mm (13%) to threshold of TST≥10 mm (10%): p = 0.12; comparing a threshold of ≥5 mm (13%) to a threshold of TST increase of ≥10 mm (8%): p = 0.02; comparing threshold of TST increase of ≥10 mm (8%) to absolute threshold of ≥10 mm(10%): p = 0.25.

The overall measured 6 month period prevalence of MTB infection was higher by QFT-GIT (38%) than by TST (33%) in the primary analysis (TST≥5 mm threshold for positivity), but this difference did not meet statistical significance (p = 0.08). When more stringent thresholds for TST positivity (i.e. TST≥10 mm at baseline or follow-up; TST≥5 mm at baseline or increase in at least 10 mm at follow-up) were considered, the estimated MTB prevalence was significantly higher for QFT-GIT (using any of the 4 proposed QFT-GIT thresholds for positivity) than for TST (Table S1).

Univariate and multivariate analysis were performed to evaluate factors associated with a positive TST or QFT-GIT (i.e. positive at either baseline or 6 months follow-up) and are found in Tables S2 and S3 (supplemental content). There was a significant association between grading of adult index case smear status and a positive QFT-GIT (AOR 6.3 [95%CI 1.1–35] and 5.4 [95%CI 1.3–23] for 2+ and 3+ smear positivity, respectively; Table S2). Index case age, gender, duration of cough, daytime duration of exposure, and nighttime exposure to pediatric contacts were not associated with a positive QFT-GIT or TST result. Of the pediatric factors that were assessed, only age >10 years was significantly associated with a positive QFT-GIT (AOR 3.8 [1.5–9.6]; p = 0.004); no pediatric factors were associated with a positive TST (Table S3).

### Analysis of QFT-GIT conversions and reversions

Among 117 children with a negative QFT-GIT at baseline and a test performed at follow-up, the follow-up test remained negative for 86 (73.5%), converted to positive for 22 (18.8%), and was indeterminate for 9 (7.7%) (Table 4). Among 62 children with a positive QFT-GIT at baseline and a test performed at follow-up, the follow-up test remained positive in 53 (85.5%) and reverted to negative in 9 (14.5%). Among 17 children with indeterminate QFT-GIT results at baseline, 13 (76%) were negative, 2 converted to positive (15%), and 2 remained indeterminate. Overall, 24/134 (18%, 95% CI [0.12–0.25]) converted their QFT-GIT from indeterminate or negative to positive (Tables 2 and 4). The mean quantitative QFT-GIT result at baseline was 4.4 IU/ml with a standard deviation of 9.5 IU/ml (median 0.18 IU/ml, IQR [0.11–1.47]), while mean QFT-GIT result at follow-up was 4.9 IU/ml with a standard deviation of 8.7 IU/ml (median 0.28, IQR [0.12–5.4]).

**Table 4:** Agreement of enrollment and 6 month follow-up results by QFT-GIT–N = 196*.

Enrollment Result	6-Month Follow-up Result	N (%, 95%CI)
Negative	Negative	86 (44, 0.37–0.51)
Negative	Positive	22 (11, 0.07–0.16)
Negative	Indeterminate	9 (5, 0.02–0.09)
Positive	Negative	9 (5, 0.02–0.09)
Positive	Positive	53 (27, 0.21–0.34)
Positive	Indeterminate	0 (0)
Indeterminate	Negative	13 (7, 0.04–0.1)
Indeterminate	Positive	2 (1, 0.001–0.04)
Indeterminate	Indeterminate	2 (1, 0.001–0.04)

*270 pediatric contacts were enrolled and 196 had follow-up visits. All 270 had baseline QFT-GIT testing. 6 month QFT-GIT testing was performed on 196 children.

In multivariate analysis of factors associated with QFT-GIT conversion at follow-up, older children were more likely to have QFT-GIT conversion than younger children (pediatric age 6–10 years AOR 6.0 [1.0–35] p = 0.048; pediatric age >10 years AOR 8.9 [1.1–72] p = 0.040, compared to pediatric age<2 years). Additionally, the rate of QFT-GIT conversions among contacts with baseline TST-positivity ≥5 mm (5/13, 38%) was higher compared to those with baseline TST-negative results (15/98[15%]; AOR 5.2 [1.2–23.4]; p = 0.03). No adult index case factors were associated with QFT-GIT conversion, including age, gender, smear status, or category of TB diagnosis. Additionally, we evaluated the impact of using three other more stringent thresholds for QFT-GIT test conversion, but we found no statistically significant difference in incidence of conversions by varying thresholds (Table 2). Overall, among children with QFT-GIT conversion (follow-up QFT-GIT >0.35 IU/ml), the mean increase was 7.5 IU/ml (median 4.07 IU/ml [IQR 1.28–10.5]).

In multivariate analysis of factors associated with QFT-GIT reversion at follow-up, female pediatric contacts (8/33; 24%) had higher rates of reversion from positive to negative QFT-GIT than males (1/29; 3.5%) (AOR 18.5 [1.1–321]; p = 0.04); pediatric contacts with a positive baseline TST (2/47[4%]) were less likely to revert from positive to negative QFT-GIT compared with those with a negative baseline TST (7/13[54%]; AOR 0.01 [0.001–0.24], p<0.01). No other index case or pediatric factors, including age, were significantly associated with QFT-GIT reversion. Overall, among those children with QFT-GIT reversions, baseline QFT-GIT results ranged from 0.66 IU/ml to 25.9 IU/ml (median 1.45 [IQR 1.2–3.5]).

### Analysis of TST conversions

Among the 196 children with 6 month follow-up, 60 children had baseline positive TST (≥5 mm) and were not retested by study protocol, while 127 had a second TST performed at six months (Table 5). Among 127 children with a negative (or unknown [n = 9]) TST at baseline, the follow-up test was negative in 110 (87.0%) and became positive (≥5 mm) in 17 (13.4%). Among these 17 TST conversions from negative (or unknown) to positive, 4 (24%) had follow-up TST between 5–9 mm, 6 (35%) had TST between 10–15 mm, and 7 (41%) had TST≥15 mm. Among these 17 TST conversions, mean baseline TST was 0.15 mm with standard deviation of 0.55 mm (median 0 mm, range 0–2 mm); mean follow-up TST was 15.7 mm with standard deviation of 8.4 mm (median 15, IQR [10–20]). When excluding those with unknown baseline TST results, 13/118 (11%) children converted from a negative baseline TST to a positive TST at 6 months. In secondary analysis, we compared different thresholds for TST conversion. Conversion rates were higher when using a simple negative to positive (i.e. from <5 mm to ≥5 mm) threshold (17/127 [13.4%]) compared to using the most stringent threshold requiring an increase of at least 10 mm induration (10/127 [7.8%]; p = .02; Table 3). Overall, mean TST response at baseline was 4.3 mm induration with a standard deviation of 7.7 mm (median 0 mm, IQR [0–5]), while mean TST response at follow-up (including baseline results if previously positive and not retested) was 6.1 mm with a standard deviation of 8.7 mm (median 0 mm, IQR [0–10]).

**Table 5:** Agreement of enrollment and 6 month follow-up results by TST–N = 194*.

Enrollment Result	6-Month Follow-up Result	N (%, 95%CI)
Negative	Negative	105 (54, 0.46–0.61)
Negative	Positive	13 (7, 0.04–0.11)
Negative	Not Available	7 (4,0.01–0.07)
Positive	Not done	60 (31, 0.24–0.38)
Not Available	Positive	4 (2,0.01–.05)
Not Available	Negative	5 (3, 0.01–.06)

*270 pediatric contacts were enrolled and 196 had follow-up visits. 254 had baseline TST testing. 6 month TST was performed on 127 children; 7 children with baseline negative TST results had study-followup, but no repeat TST results were available; 60 children with follow-up visits had baseline TST≥5 mm and were not retested. 74 children had no follow-up data.

In multivariate regression analysis, only baseline QFT-GIT positivity was associated with converting TST from negative to positive (AOR 11.1 [1.6–76.6]; p = 0.01). The rate of TST conversion (from <5 mm to ≥5 mm) among contacts that were QFT-GIT-positive/TST-negative at baseline was 46% (6/13), compared to 7% (6/92) among those with concordant QFT-GIT-negative/TST-negative baseline results. No index case factors or other pediatric factors were significantly associated with TST conversion in multivariate regression analysis.

### Concordance of TST and QFT conversions

Concordance between TST and QFT-GIT conversions is shown in Table S4. The percent agreement in TST and QFT-GIT conversions ranged from 83%–85%, though kappa values suggested only ‘fair’ agreement (range 0.11–0.33). The definition of QFT-GIT conversion had little impact on concordance with TST conversion. The highest concordance between tests was seen when using the less stringent TST threshold of going from a baseline of <5 mm induration to ≥5 mm induration, cross-tabulated with any of the four proposed thresholds for QFT-GIT conversion.

### TB status and treatment among pediatric contacts at 6 months follow-up

At the 6-month visit, only 57/196 (29%) children were reported by their parent/guardian to have been started on any antimycobacterial treatment since the baseline visit. Among those started on antimycobacterial therapy, 26/57 (46%) were positive at baseline by both TST (5 mm threshold) and QFT-GIT; 7/57 (12%) were TST-positive and QFT-GIT- negative or indeterminate; 7/57 (12%) were TST-negative and QFT-GIT-positive; and 17 (29%) were TST-negative with either a negative or indeterminate QFT-GIT. Eighteen of 57 (31.5%) were on isoniazid monotherapy and 39/57 (68.4%) were on multidrug treatment for presumed active TB. No microbiological results were available for any children.

There did not appear to be any significant association between receipt of anti-tuberculous treatment and TST or QFT-GIT conversions or reversions. Among 134 children with a baseline negative/indeterminate QFT-GIT, 20/110 (18%) not receiving any treatment had QFT-GIT conversions, while 4/24 (17%) receiving anti-tuberculous treatment had QFT-GIT conversions (p = 0.861). Among 62 children with a positive baseline QFT-GIT and follow-up testing available, 3/29 (10%) not receiving any treatment reverted to a negative QFT-GIT result, while 6/33 (18%) receiving treatment reverted to a negative QFT-GIT result (p = 0.382). Among 118 children with negative baseline TST (<5 mm), 9/97 (9%) not receiving any treatment converted their TST to positive, while 4/21 (19%) receiving anti-tuberculous treatment had TST conversions (p = 0.195).

## Discussion

Identifying pediatric TB infection remains challenging, and children who are contacts of adult household TB cases are at particularly high risk for infection. Consequently, the national TB program in South Africa recommends active contact investigations for children who are household contacts of adult pulmonary TB cases [5]. We explored and compared the utility of follow-up TB testing among these pediatric contacts using TST and QFT-GIT, and found high TB prevalence rates. Importantly, we found that the prevalence estimates of TB infection in these children increased significantly at 6 months compared to baseline, regardless of choice of test. When using the QFT-GIT test, prevalence estimates increased from 29% at baseline to 38% by 6 months; similarly, prevalence increased from 28% to 33% when using TST. These results suggest that serial testing among pediatric contacts of adult TB cases has a higher yield in detecting TB infection than single tests. It could not be ascertained from our study whether test conversions from baseline to 6 months among these children is the result of delayed development of immunologic responses in children, or from ongoing household or community TB exposure. However, our results underscore the fact that limiting contact investigations to the time period immediately after index case diagnosis may miss cases of TB infection among pediatric contacts. While the optimal time period for follow-up testing was not evaluated in this study, our results suggest that serial testing six months after the initial contact investigation may identify an increased number of pediatric contacts with *M. tuberculosis* infection, compared to more limited contact investigations.

Overall, we found that the 6-month prevalence of *M. tuberculosis* infection among pediatric contacts was similar as ascertained by QFT-GIT and TST (5 mm induration cut-off). However, we found that significantly more children would be identified with TB infection by QFT-GIT than TST when more stringent thresholds for TST positivity and conversion were used. In contrast, there was little change in overall QFT-GIT prevalence results when using alternative higher (more stringent) thresholds for QFT-GIT test conversion, and quantitative QFT-GIT changes were robust (mean increase 7.5 IU/ml). Concordance was highest between the two tests when using the least stringent definition of TST conversion (i.e. simple negative to positive change at 5 mm induration). These observations are consistent with recently published data suggesting impaired sensitivity of TST when using higher thresholds for positivity (i.e. >10 mm or 15 mm induration) among children at risk for TB infection [15], [16]. These results may indicate that IGRAs are more sensitive at identifying new infections compared to TST when higher cut-offs are used to define TST positivity.

To date, there has been limited published information on serial TB testing using IGRAs in children. Moreover, there has been no consensus on whether the same cut-offs that are used for latent TB diagnosis should also be used to define an IGRA conversion. Our results provide important insight into QFT-GIT performance in a high risk, heavily-exposed population that will allow better understanding and interpretation of test results in children. Similar studies in adult populations have suggested that IGRA conversion rates may be dependent on the definitions and thresholds utilized to define conversion [8], [17]. In contrast, we did not find a similar phenomenon in our population of pediatric TB contacts, even when using more stringent criteria to define QFT-GIT conversion. This finding leads us to postulate that these conversions in our study were more likely to be the result of new TB infection rather than within-person variability in IGRA responses. On the other hand, prevalence and conversion rates ascertained by TST were dependant on the definitions used to define positivity, making interpretation of serial TST testing results in the context of contact investigations more challenging.

Interestingly, we found that baseline TST and QFT-GIT discordance was associated with test conversions on follow-up testing. Children who were TST-positive but QFT-GIT-negative at baseline were more likely to convert to a positive QFT-GIT result compared to those that were concordant TST and QFT-GIT negative. Similarly, children with QFT-GIT-positive/TST-negative discordance at baseline were more likely to convert to a positive TST result at follow-up than those that were TST-negative/QFT-GIT-negative at baseline. These findings underscore the challenge of interpreting these tests in the absence of a reference standard for diagnosing latent TB infection, and suggest that neither test likely has ideal sensitivity.

IGRA reversions to negative have been previously reported and we similarly found a relatively high rate of QFT-GIT reversions (15%) among pediatric household TB contacts [10], [17], [18]. Previously, it has been suggested that some reversions may be accounted for by minor variations in IGRA responses around the cut-off points for positivity [8]. However, we found no reversions among children with weakly positive baseline QFT-GIT results, and all observed reversions in our study occurred in children with robust baseline IGRA responses (median 1.5 IU/ml). We found an association between female gender and reversion of QFT-GIT results, which has not been previously reported, but it remains to be seen if this finding is replicated in other settings. Children who were QFT-GIT positive at baseline were also more likely to revert to a negative QFT-GIT result at follow-up if they had a baseline negative TST. Some have previously reported reversions or reductions in IGRA responses following latent TB treatment [19], [20]. In contrast, we did not find a significant association between receipt of anti-tuberculous treatment and reversion of IGRA responses. Ultimately, whether QFT-GIT reversion represents impaired specificity, clearing of *M. tuberculosis* infection or reduced secretion of mycobacterial antigens, or a transient variability in immunologic responses is unclear and warrants further study.

Our study has several limitations. Six month follow-up test data was only available on approximately 75% of children that were initially enrolled. Our prevalence results therefore likely represent an underestimation of the true burden of *M. tuberculosis* infection in this population. Additionally, children with a positive TST result at baseline did not receive a follow-up TST, and therefore we could not explore factors associated with TST reversions. Also, there was a higher rate of HIV infection among children lost to follow-up (9%) compared to those with serial testing available (4%), suggesting that the study may have excluded a subgroup of sicker children. This potential selection bias could lead to underestimation of the prevalence of *M. tuberculosis* infection in this population. Finally, we did not have access to microbiological information on study participants, and were therefore unable to correlate TST and QFT-GIT results with mycobacterial culture data. Nonetheless, we report treatment information for children who presented for 6-month follow-up visits and explored the association of MTB treatment with TST and QFT-GIT positivity.

In conclusion, the estimated prevalence of TB infection in South Africa among pediatric contacts of adult household cases was high using a follow-up test six months later and a strategy for contact investigations that incorporates follow-up testing may be advisable. Serial testing is feasible and useful in this setting, and IGRAs may represent an alternative testing option to TST. IGRA conversions and reversions occur frequently on serial-testing and further studies are warranted to determine the long-term prognosis of children who exhibit these test patterns.

## Supporting Information

Figure S1 **Flow diagram for study participants.**
(TIF)

Table S1 Comparison of MTB prevalence estimates by QFT-GIT and TST.
(DOC)

Table S2 Adult index case factors associated with a positive QFT-GIT or TST by 6 months. *Positivity defined as QFT-GIT (≥0.35 IU/ml) or TST (≥5 mm) at baseline and/or 6-month follow-up. Other factors considered in the univariate analysis that were not statistically significant and not shown in the tables included nighttime exposure to index case, number of bedrooms in house, number of adults living in the household, and duration of cough for the adult. The associations found in regression analysis between index case and pediatric factors and QFT-GIT positivity did not change when using any of the alternative thresholds for QFT-GIT conversion. Similarly, the associations found in regression analysis between index case factors and TST did not change when considering higher thresholds for TST positivity. † p = 0.01 (QFT-GIT), p = 0.009 (TST); ** p = 0.037, 0.027, <0.0001, respectively for 2+, and 3+; “scanty” dropped from the regression; ‡ p = 0.011 for 3+ respectively; ‡‡ p = 0.002, 0.002 for “Smear-negative, Culture-positive” and “Clinical TB”, respectively; *** p = 0.002.
(DOC)

Table S3 Pediatric factors associated with a QFT-GIT or TST by 6 months. * A TST induration of 5 mm was used as the cut-off point for test positivity. Other factors considered in the univariate analysis that were not statistically significant and not shown in the tables included height for age Z score and weight for age Z score. In multivariate analysis using a higher threshold for TST positivity (TST≥10 mm), pediatric age >10 yrs was significantly associated with a positive TST (aOR 3.5 [95%CI 1.2–10]; p = 0.02). Similarly age >10 yrs was significantly associated with a positive result when using the threshold of a TST increase of at least 10 mm at follow-up(aOR 3.1 [95%CI 1.1–8.3]; p = 0.03). † p = 0.007; ** P = .004.
(DOC)

Table S4 Concordance between TST and QFT conversions among children with concordant negative results at baseline (n = 114)*. *Includes those with indeterminate baseline QFT-GIT/negative TST.
(DOC)
