# Supplementary figures and images for: Longitudinal Analysis of QuantiFERON-TB Gold In-Tube in Children with Adult Household Tuberculosis Contact in South Africa: A Prospective Cohort Study
Source: PLoS One. 2011 Oct 31;6(10):e26787. doi: 10.1371/journal.pone.0026787 (PMC3204993; doi:10.1371/journal.pone.0026787)

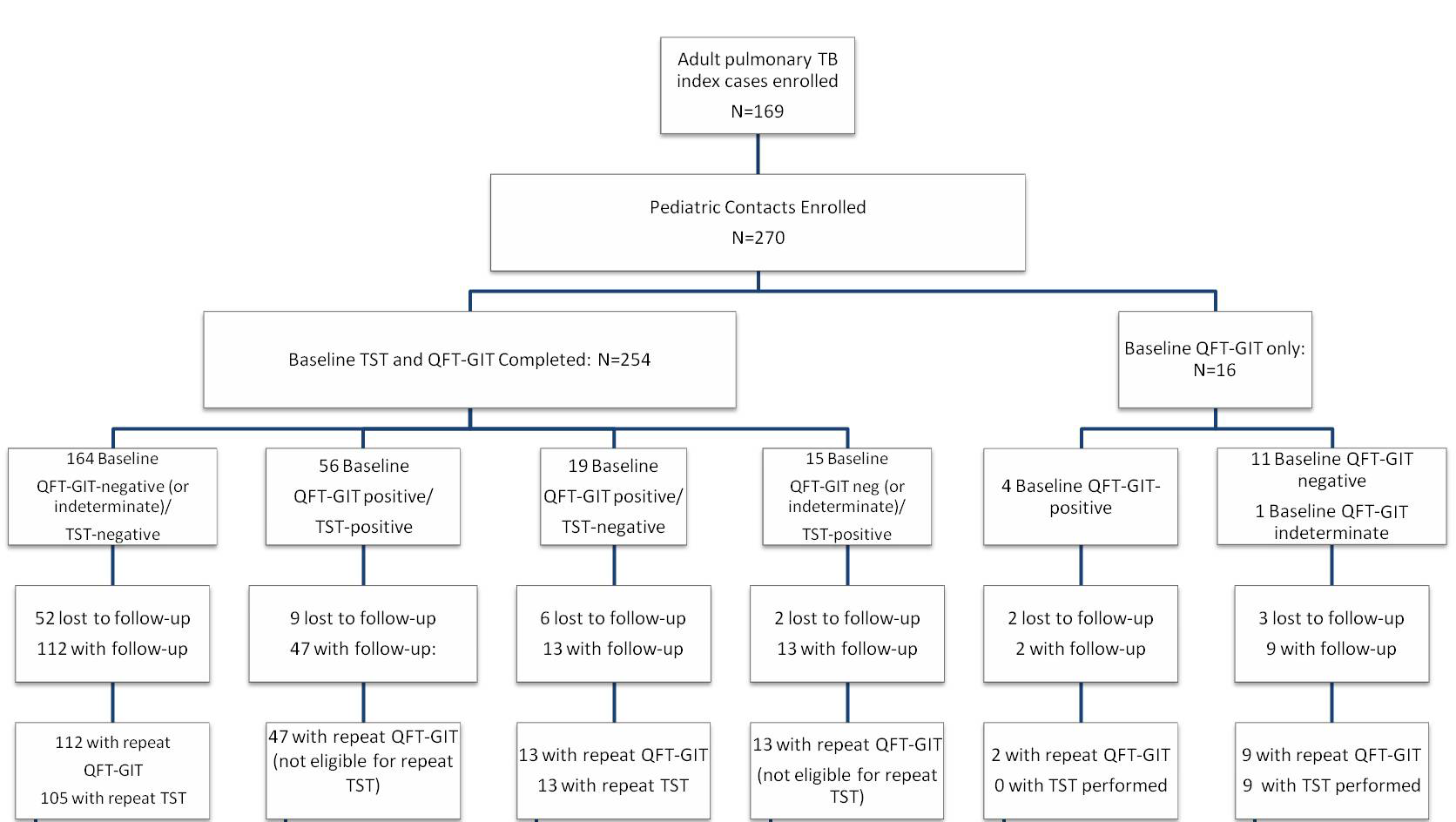

Supplement: Figure S1 — Flow diagram for study participants. (TIF) [file pone.0026787.s001.tif]
