# Supplementary material for: Longitudinal Analysis of QuantiFERON-TB Gold In-Tube in Children with Adult Household Tuberculosis Contact in South Africa: A Prospective Cohort Study
Source: PLoS One. 2011 Oct 31;6(10):e26787. doi: 10.1371/journal.pone.0026787 (PMC3204993; doi:10.1371/journal.pone.0026787)
Supplement: Table S1 — Comparison of MTB prevalence estimates by QFT-GIT and TST. (DOC) [file pone.0026787.s002.doc]

**Supplemental Table 1: Comparison of MTB prevalence estimates by QFT-GIT and TST**

|  |  |  | Six month MTB prevalence estimates by different  TST thresholds for positivity | | |
| --- | --- | --- | --- | --- | --- |
|  |  |  | Baseline or  6 month  TST ≥ 5mm | Baseline or 6 month  TST ≥ 10mm | Baseline TST ≥ 5mm or 6 month TST with ≥10mm increase from baseline |
|  |  |  | 88/263 (33%) | 70/263 (27%) | 81/263 (31%) |
|  | Baseline or 6 month  QFT-GIT ≥0.35 IU/ml | 103/270 (38%) | P=0.08 | P<0.01 | P=0.01 |
| Six month MTB prevalence estimates by different | Baseline QFT-GIT ≥0.35 IU/ml or  6 month QFT-GIT ≥0.35 IU/ml, plus 30% increase over baseline | 103/270 (38%) | P=0.08 | P<0.01 | P=0.01 |
| QFT-GIT threshold for positivity | Baseline QFT-GIT ≥0.35 IU/ml or  6 month QFT-GIT ≥0.35 IU/ml, plus absolute increase of 0.35 IU/ml over baseline | 102/270 (38%) | P=.12 | P<0.01 | P=0.01 |
|  | Baseline QFT-GIT ≥0.35 IU/ml or  6 month Repeat QFT-GIT ≥0.70 IU/ml | 102/270 (38%) | P=.12 | P<0.01 | P=0.01 |
