# Supplementary material for: Longitudinal Analysis of QuantiFERON-TB Gold In-Tube in Children with Adult Household Tuberculosis Contact in South Africa: A Prospective Cohort Study
Source: PLoS One. 2011 Oct 31;6(10):e26787. doi: 10.1371/journal.pone.0026787 (PMC3204993; doi:10.1371/journal.pone.0026787)
Supplement: Table S2 — Adult index case factors associated with a positive QFT-GIT or TST by 6 months. *Positivity defined as QFT-GIT (≥0.35 IU/ml) or TST (≥5 mm) at baseline and/or 6-month follow-up. Other factors considered in the univariate analysis that were not statistically significant and not shown in the tables included nighttime exposure to index case, number of bedrooms in house, number of adults living in the household, and duration of cough for the adult. The associations found in regression analysis between index case and pediatric factors and QFT-GIT positivity did not change when using any of the alternative thresholds for QFT-GIT conversion. Similarly, the associations found in regression analysis between index case factors and TST did not change when considering higher thresholds for TST positivity. † p = 0.01 (QFT-GIT), p = 0.009 (TST); ** p = 0.037, 0.027, <0.0001, respectively for 2+, and 3+; “scanty” dropped from the regression; ‡ p = 0.011 for 3+ respectively; ‡‡ p = 0.002, 0.002 for “Smear-negative, Culture-positive” and “Clinical TB”, respectively; *** p = 0.002. (DOC) [file pone.0026787.s003.doc]

**Supplemental Table 2. Adult index case factors associated with a positive QFT-GIT or TST by 6 months**

|  | | | | | | |
| --- | --- | --- | --- | --- | --- | --- |
|  | **QFT-GIT*** | | | **TST (5mm cut-off for positivity)*** | | |
|  | **n positive/total (%)** | **OR (95% CI)** | **AOR (95% CI)** | **n positive/total (%)** | **OR (95% CI)** | **AOR (95% CI)** |
| Adult index case age (years)  18-25  26-35  36-45  >45 | 11/23(48)  44/130(34)  35/90(39)  13/27(48) | REF  0.56 (0.21-1.5)  0.69 (0.25-1.9)  1.0 (0.31-3.2) | REF  0.89 (0.25-3.2)  1.2 (0.31-4.9)  2.2 (0.44-10.5) | 11/22 (50)  39/128 (30)  30/88 (34)  8/25 (32) | REF  0.44 (0.15-1.3)  0.52(0.17-1.6)  0.47(0.11-2.0) | REF  0..57 (0.15-2.1)  1.2 (0.30-4.5)  0.97 (0.15-6.5) |
| Adult index case sex  Male  Female | 28/73 (38)  75/197(38) | REF  0.99 (0.5-1.8) | REF  1.5 (0.68-3.3) | 24/71 (34)  64/192 (33) | REF  0.98 (0.51-1.9) | REF  1.5 (0.60-3.4) |
| Adult index case HIV status  Infected  Uninfected | 92/249 (37)  5/7 (71) | 0.23 (0.23-2.6)  REF | 0.36 (0.04-3.7)  REF | 76/242(31)  5/7(71) | 0.18 (0.02-1.9)  REF | 0.30 (0.30-3.1)  REF |
| Adult index case type of TB diagnosis:  Smear positive TB  Smear negative, culture positive TB  Clinical TB | 50/113 (44)  6/34 (18)  47/123 (38) | REF  **0.27 (0.09-0.8)** †  078 (0.45-1.4) | REF  0.60 (0.1-3.1)  1.6 (0.41-6.5) | 48/110 (44)  5/33 (15)  35/120 (23) | REF  **0.23 (0.08-0.7) †**  0.53 (0.28-1.01) | REF  0.**09 (0.02-0.4)‡‡**  **0.15 (0.05-0.51)‡‡** |
| Adult index case smear grade  Negative  Scanty  1+  2+  3+ | 34/115 (30)  3/12 (25)  19/50 (38)  9/19 (47)  19/32 (59) | REF  0.79 (0.2-2.9)  1.5 (0.7-3.2)  2.1 (0.6-7.2)  **3.4 (1.6-7.7)***** | REF  --  2.2 (0.55-9.1)  **6.3 (1.1-35)****  **5.4 (1.3-23)**** | 28/112 (25)  3/12 (25)  19/49 (39)  9/18 (50)  17/31 (54) | REF  1.0 (.1-9.8)  1.9 (.8-4.4)  3.0 (0.8-10.8)  **3.6 (1.3-9.9)‡** | REF  0.12 (0.01-1.5)  0.31(0.08-1.2)  --  0.64(0.17-2.4) |
| Exposure to Index Case During the Day  Minority of day (< 6 hours)  Majority of day (> 7 hours) | 44/113 (39)  58/154 (38) | REF  .95 (0.56-1.6) | REF  1.1 (0.6-1.9) | 35/111 (31)  53/149 (36) | REF  1.2 (0.7-2.1) | REF  1.3 (0.68-2.3) |

**Legend for Supplemental Table 2**

*positivity defined as QFT-GIT (≥0.35 IU/ml) or TST (≥5mm) at baseline and/or 6-month follow-up. Other factors considered in the univariate analysis that were not statistically significant and not shown in the tables included nighttime exposure to index case, number of bedrooms in house, number of adults living in the household, and duration of cough for the adult. The associations found in regression analysis between index case and pediatric factors and QFT-GIT positivity did not change when using any of the alternative thresholds for QFT-GIT conversion. Similarly, the associations found in regression analysis between index case factors and TST did not change when considering higher thresholds for TST positivity.

† p=0.01 (QFT-GIT), p=0.009 (TST)

** p=0.037, 0.027, <0.0001, respectively for 2+, and 3+; “scanty” dropped from the regression

‡ p=0.011 for 3+ respectively;

‡‡ p=0.002, 0.002 for “Smear-negative, Culture-positive” and “Clinical TB”, respectively

*** p=0.002
