# Supplementary material for: Longitudinal Analysis of QuantiFERON-TB Gold In-Tube in Children with Adult Household Tuberculosis Contact in South Africa: A Prospective Cohort Study
Source: PLoS One. 2011 Oct 31;6(10):e26787. doi: 10.1371/journal.pone.0026787 (PMC3204993; doi:10.1371/journal.pone.0026787)
Supplement: Table S3 — Pediatric factors associated with a QFT-GIT or TST by 6 months. * A TST induration of 5 mm was used as the cut-off point for test positivity. Other factors considered in the univariate analysis that were not statistically significant and not shown in the tables included height for age Z score and weight for age Z score. In multivariate analysis using a higher threshold for TST positivity (TST≥10 mm), pediatric age >10 yrs was significantly associated with a positive TST (aOR 3.5 [95%CI 1.2–10]; p = 0.02). Similarly age >10 yrs was significantly associated with a positive result when using the threshold of a TST increase of at least 10 mm at follow-up(aOR 3.1 [95%CI 1.1–8.3]; p = 0.03). † p = 0.007; ** P = .004. (DOC) [file pone.0026787.s004.doc]

|  | **QFT-GIT*** | | | **TST (5mm cut-off)*** | | |
| --- | --- | --- | --- | --- | --- | --- |
|  | **n positive/total (%)** | **OR (95% CI)** | **AOR (95% CI)** | **n positive/total (%)** | **OR (95% CI)** | **AOR (95% CI)** |
| Pediatric contact age (years)  <2  2-5  6-10  >10 | 12/51 (23)  29/76 (38)  35/91 (38)  27/52 (52) | REF  2.0 (0.94-4.3)  2.0 (0.94-4.4)  **3.5 (1.4-8.7) †** | REF  2.1 (0.94-4.6)  2.0 (0.91-4.2)  **3.8 (1.5-9.5)**** | 14/48 (29)  29/74 (39)  22/89 (24)  23/52 (44) | REF  1.6 (0.72-3.4)  0.80 (0.37-1.7)  1.9 (0.81-4.5) | REF  1.5 (0.68-3.5)  0.89 (0.39-2.0)  2.3 (0.24-3.4) |
| Pediatric Contact Sex  Male  Female | 50/129 (39)  53/141 (38) | REF  0.95 (0.56-1.6) | REF  0.90 (0.51-1.6) | 40/123 (32)  48/140 (34) | REF  1.1 (0.61-1.9) | REF  1.0 (0.57-1.8) |
| Pediatric contact HIV status  Infected  Uninfected | 6/14 (43)  95/251 (38) | 1.2 (0.41-3.7)  REF | 1.6 (0.40-5.9)  REF | 4/14 (29)  81/244 (33) | 0.80 (0.24-2.7)  REF | 0.93(0.24-3.6)  REF |
| Pediatric contact BCG vaccination status  Vaccinated  Not Vaccinated | 98/257 (29)  3/5 (40) | REF  2.4 (0.60-9.9) | REF  1.8 (0.4-8.0) | 83/250 (33)  2/5 (40) | REF  1.4 (0.17-10) | REF  0.90 (0.57-1.8) |
| Pediatric contact weight for height Z score  Z<-2  -2>z<2  Z>2 | 1/3 (33)  25/71 (35)  7/29 (24) | 0.92 (0.08-11)  REF  0.58(0.22-1.6) | -- | 0/2 (0)  28/71 (39)  9/27 (33) | --  REF  0.7 (0.3-1.9) | -- |

**Supplemental Table 3. Pediatric factors associated with a QFT-GIT or TST by 6 months**

**Legend for Supplemental Table 3**

* A TST induration of 5 mm was used as the cut-off point for test positivity. Other factors considered in the univariate analysis that were not statistically significant and not shown in the tables included height for age Z score and weight for age Z score. In multivariate analysis using a higher threshold for TST positivity (TST≥10mm), pediatric age >10 yrs was significantly associated with a positive TST (aOR 3.5 [95%CI 1.2-10]; p=0.02). Similarly age >10 yrs was significantly associated with a positive result when using the threshold of a TST increase of at least 10mm at follow-up(aOR 3.1 [95%CI 1.1-8.3]; p=0.03).

† p=0.007

** P=.004
