# Supplementary material for: Longitudinal Analysis of QuantiFERON-TB Gold In-Tube in Children with Adult Household Tuberculosis Contact in South Africa: A Prospective Cohort Study
Source: PLoS One. 2011 Oct 31;6(10):e26787. doi: 10.1371/journal.pone.0026787 (PMC3204993; doi:10.1371/journal.pone.0026787)
Supplement: Table S4 — Concordance between TST and QFT conversions among children with concordant negative results at baseline (n = 114)*. *Includes those with indeterminate baseline QFT-GIT/negative TST. (DOC) [file pone.0026787.s005.doc]

**Supplemental Table 4: Concordance between TST and QFT conversions among children with concordant negative results at baseline (n=114)***

|  | | | | | | |
| --- | --- | --- | --- | --- | --- | --- |
|  | TST threshold for conversion at follow-up | | | | | |
| QFT-GIT threshold for conversion at follow-up | Baseline TST <5m,  repeat TST ≥5mm | | Baseline TST <10mm,  repeat TST ≥10mm | | Baseline TST <5mm,  Repeat TST increase of 10mm | |
|  | Concordance | Kappa | Concordance | Kappa | Concordance | Kappa |
| QFT-GIT ≥0.35 IU/ml | 85% | .33 | 84% | .23 | 83% | .11 |
| QFT-GIT ≥0.35 IU/ml,  plus 30% increase over baseline | 85% | .33 | 84% | .23 | 83% | .11 |
| QFT-GIT ≥0.35 IU/ml, plus absolute increase of 0.35IU/ml over baseline | 85% | .33 | 84% | .23 | 83% | .11 |
| QFT-GIT ≥0.70 IU/ml | 85% | .33 | 84% | .23 | 83% | .11 |

**Legend for Supplemental Table 4:**

*Includes those with indeterminate baseline QFT-GIT/negative TST
